# Long-term safety and efficacy of vismodegib in patients with advanced basal cell carcinoma: final update of the pivotal ERIVANCE BCC study

**DOI:** 10.1186/s12885-017-3286-5

**Published:** 2017-05-16

**Authors:** Aleksandar Sekulic, Michael R. Migden, Nicole Basset-Seguin, Claus Garbe, Anja Gesierich, Christopher D. Lao, Chris Miller, Laurent Mortier, Dedee F. Murrell, Omid Hamid, Jorge F. Quevedo, Jeannie Hou, Edward McKenna, Natalie Dimier, Sarah Williams, Dirk Schadendorf, Axel Hauschild, for the ERIVANCE BCC Investigators

**Affiliations:** 1 Mayo Clinic Scottsdale, 13400 East Shea Boulevard, Scottsdale, AZ 85259 USA; 2 Departments of Dermatology and Head and Neck Surgery, The University of Texas MD Anderson Cancer Center, 1400 Pressler Street, Houston, TX 77030 USA; 3 Service de Dermatologie, Hôpital Saint-Louis, 1 av claude Vellefaux, 75010 Paris, France; 4 Studienzentrum Dermatologische Onkologie, Universitätsklinikum Tübingen, Liebermeisterstr. 25, 72074 Tübingen, Germany; 5 Klinik für Dermatologie, Venerologie und Allergologie, Universitätsklinikum Würzburg, Josef-Schneider-Str. 2, 97080 Würzburg, Germany; 6 University of Michigan, 1500 East Medical Center Drive, Ann Arbor, MI 48109 USA; 7 Department of Dermatology, University of Pennsylvania Medical Center, 3400 Civic Center Boulevard, Philadelphia, PA 19104 USA; 8 Clinique de Dermatologie, Hôpital Claude Huriez, Inserm U1189, Lille, France; 9 Dermatology Department, St George Clinical School, University of New South Wales, Grey Street, Sydney, 2217 Australia; 10 The Angeles Clinic and Research Institute, 1818 Wilshire Boulevard, Los Angeles, California USA; 11 Mayo Clinic, 200 First Street SW, Rochester, MN 55905 USA; 12 Genentech, Inc., 1 DNA Way, South San Francisco, California 94080 USA; 13 Roche Products Limited, Hexagon Place, 6 Falcon Way, Shire Park, Welwyn Garden City, Hertfordshire Al7 1TW UK; 14 Klinikum für Dermatologie, Venerologie und Allergologie, Universitätsklinikum Essen, Hufelandstrabe 55, 45147 Essen, Germany; 15 Universitätsklinikum Schleswig-Holstein, Schittenhelmstr, 7, D-24 105 Kiel, Germany

**Keywords:** Basal cell carcinoma (BCC), Vismodegib, Long-term, Safety, Efficacy

## Abstract

**Background:**

In the primary analysis of the ERIVANCE BCC trial, vismodegib, the first US Food and Drug Administration–approved Hedgehog pathway inhibitor, showed objective response rates (ORRs) by independent review facility (IRF) of 30% and 43% in metastatic basal cell carcinoma (mBCC) and locally advanced BCC (laBCC), respectively. ORRs by investigator review were 45% (mBCC) and 60% (laBCC). Herein, we present long-term safety and final investigator-assessed efficacy results in patients with mBCC or laBCC.

**Methods:**

One hundred four patients with measurable advanced BCC received oral vismodegib 150 mg once daily until disease progression or intolerable toxicity. The primary end point was IRF-assessed ORR. Secondary end points included ORR, duration of response (DOR), progression-free survival, overall survival (OS), and safety.

**Results:**

At data cutoff (39 months after completion of accrual), 8 patients were receiving the study drug (69 patients in survival follow-up). Investigator-assessed ORR was 48.5% in the mBCC group (all partial responses) and 60.3% in the laBCC group (20 patients had complete response and 18 patients had partial response). ORRs were comparable across patient subgroups, including aggressive histologic subtypes (eg, infiltrative BCC). Median DOR was 14.8 months (mBCC) and 26.2 months (laBCC). Median OS was 33.4 months in the mBCC cohort and not estimable in the laBCC cohort. Adverse events remained consistent with clinical experience. Thirty-three deaths (31.7%) were reported; none were related to vismodegib.

**Conclusions:**

This long-term update of the ERIVANCE BCC trial demonstrated durability of response, efficacy across patient subgroups, and manageable long-term safety of vismodegib in patients with advanced BCC.

**Trial registration:**

This study was registered prospectively with Clinicaltrials.gov, number NCT00833417 on January 30, 2009.

**Electronic supplementary material:**

The online version of this article (doi:10.1186/s12885-017-3286-5) contains supplementary material, which is available to authorized users.

## Background

Basal cell carcinoma (BCC), the most common human malignancy [1, 2], presents a significant public health burden [3]. Although BCC can be treated with surgery or radiation therapy in the majority of cases, disease may progress to become locally advanced (laBCC) or, rarely, metastatic (mBCC) [4]. It is estimated that up to one-third of giant BCCs occur in the setting of delay in diagnosis and treatment [5]. Such patients may no longer be responsive to conventional therapy [6–8] and face limited options. The majority of BCC tumors, including laBCC and mBCC, harbor genetic alterations in the Hedgehog signaling pathway, leading to abnormal pathway activation and uncontrolled cellular proliferation [9, 10]. As the principal driver in BCC pathogenesis and progression, the Hedgehog pathway represents a key therapeutic target. Vismodegib, a first-in-class small molecule inhibitor of Hedgehog pathway signaling [11–13], was approved by the US Food and Drug Administration (FDA) and the European Medicines Agency (EMA) for the treatment of adults with mBCC or with laBCC that has recurred after surgery or who are not candidates for surgery or radiation [14, 15]. Vismodegib is currently approved in more than 60 countries worldwide. More recently, a second Hedgehog pathway inhibitor (HPI) (sonidegib) has been approved for laBCC based on the results from the BOLT study [16].

Primary analysis of the pivotal phase II ERIVANCE BCC trial of vismodegib [17] met its primary end point, with an independent review facility (IRF)–assessed response rate of 30% in patients with mBCC and 43% in patients with laBCC and an investigator-assessed median duration of response (DOR) of 12.9 and 7.6 months, respectively. The primary analysis was conducted 9 months after completion of accrual. As vismodegib is the first HPI in wide clinical use, and because some patients with advanced BCC, including patients with Gorlin syndrome (also known as basal cell carcinoma nevus syndrome [BCCNS]), may require prolonged vismodegib treatment, it is particularly important to understand the safety and efficacy of long-term vismodegib therapy. Here, we report final data from ERIVANCE BCC, with 39 months of follow-up after the completion of accrual, that confirms and extends the long-term safety and durability of response associated with vismodegib and further evaluates efficacy across relevant patient subgroups and tumor histologic subtypes.

## Methods

### Patient eligibility

Patient eligibility criteria have been previously described [17]. Eligible patients with mBCC had histologic confirmation of metastatic disease that was measurable according to Response Evaluation Criteria In Solid Tumors, version 1.0 (RECIST v1.0) [18], as assessed by computed tomography or magnetic resonance imaging. Patients with laBCC had at least 1 histologically confirmed lesion of ≥10 mm in the longest diameter that had recurred after radiotherapy (unless radiotherapy was contraindicated or inappropriate) and for which curative surgery was not possible, medically contraindicated, or inappropriate in the opinion of a Mohs dermatologic surgeon, head and neck surgeon, or plastic surgeon. Acceptable medical contraindications to surgery included anticipated substantial morbidity and/or deformity from surgery (eg, removal of all or part of a facial structure, such as nose, ear, eyelid, or eye; or requirement for limb amputation). Patients with Gorlin syndrome were eligible for enrollment, provided they met all other inclusion criteria [17].

### Study design

This was a phase II, single-arm, 2-cohort, multicenter study to evaluate the efficacy and safety of vismodegib in patients with advanced BCC (Clinicaltrials.gov: NCT00833417) [17]. The study was conducted in accordance with FDA regulations and the ethical principles of the Declaration of Helsinki, and within the International Conference on Harmonization E6 Guideline for Good Clinical Practice. Before study initiation, the protocol was approved by an independent review board or ethics committee at each study site. All patients provided written informed consent.

All patients received oral vismodegib 150 mg once daily until disease progression, unacceptable toxicity, or withdrawal from the study. The primary end point was objective response rate (ORR), determined by an IRF. Secondary end points included investigator-assessed ORR, IRF- and investigator-assessed DOR, progression-free survival (PFS), overall survival (OS), and change from day 1 in patient-reported symptoms, safety, and absence of residual BCC in the laBCC cohort. For this final update, all assessments were made by investigators in the efficacy-evaluable population.

### Analysis and assessments

Patients with independently confirmed BCC pathology were considered evaluable for efficacy. In the mBCC cohort, tumor responses were evaluated radiologically according to RECIST v1.0. Tumor response in the laBCC cohort was assessed using a composite end point: a decrease of ≥30% in the externally visible or radiographic dimension or complete resolution (re-epithelialization) of ulceration (if present at baseline). Response was defined as complete response (CR) in the absence of residual BCC in a tumor biopsy specimen obtained at week 24 or at best response, or partial response (PR) determined by 2 consecutive assessments performed ≥4 weeks apart. Progressive disease was defined as an increase of ≥20% in the externally visible or radiographic dimension or the presence of new ulceration or lesions. In cases in which lesion borders were indiscernible, the scar was included in the tumor measurement. Tumors were assessed at baseline and at 8-week intervals. The Kaplan–Meier method was used to estimate the median DOR, PFS, and OS, with censoring of patients who had not experienced events at the time of the last tumor assessment (DOR and PFS) or last patient contact (OS).

All treated patients were considered evaluable for safety. Safety analyses included frequency and severity of treatment-emergent adverse events (TEAEs), AEs leading to treatment interruption or discontinuation, serious AEs (SAEs), and death. AEs were graded according to National Cancer Institute Common Terminology Criteria for Adverse Events, version 3.0 [19].

Exploratory post hoc analyses were conducted to assess ORR by baseline characteristics, including Eastern Cooperative Oncology Group (ECOG) performance status, age, region, sex, ethnicity, number of target lesions, and histologic subtype using descriptive statistical methods. Additionally, exploratory analyses were conducted to evaluate the impact of missed vismodegib doses on ORR and to assess the incidence of TEAEs according to duration of treatment (<12 months vs ≥12 months) with vismodegib.

## Results

### Baseline characteristics and patient disposition

A total of 104 patients, 33 with mBCC and 71 with laBCC, were enrolled at 31 sites in the United States, Europe, and Australia. Baseline patient and disease characteristics have been presented previously [17]. At the time of this data cutoff, 39 months after the completion of accrual, 8 patients (8%) were continuing to receive treatment with vismodegib and to undergo protocol-specified assessments, while 69 patients (66%) remained in survival follow-up. Treatment had been discontinued in 96 patients, primarily because of disease progression (27.9%), patient decision to withdraw (26.0%), and AEs (21.2%). Patient disposition is shown in Table 1.

**Table 1:** Patient disposition: long-term analysis

Disposition, *n* (%)	mBCC	laBCC	All patients
	(*n* = 33)	(*n* = 71)	(*N* = 104)
On treatment	1 (3.0)	7 (9.9)	8 (7.7)
Discontinued treatment	32 (97.7)	64 (90.1)	96 (92.3)
Main reason			
AE	5 (15.2)	17 (23.9)	22 (21.2)
Death	1 (3.0)	2 (2.8)	3 (2.9)
Lost to follow-up	1 (3.0)	2 (2.8)	3 (2.9)
Physician decision	3 (9.1)	7 (9.9)	10 (9.8)
Patient decision	4 (12.1)	23 (32.4)	27 (26.0)
Disease progression	17 (51.5)	12 (16.9)	29 (27.9)
Other	1 (3.0)	1 (1.4)	2 (1.9)

*AE* adverse event, *laBCC* locally advanced basal cell carcinoma, *mBCC* metastatic basal cell carcinoma

### Treatment exposure

Median duration (range) of treatment with vismodegib was 12.9 (0.7–47.8) months (13.3 [0.7–39.1] months in the mBCC cohort and 12.7 [1.1–47.8] months in the laBCC cohort). Overall median dose intensity achieved by patients while on treatment was 97.4% (98.9% and 96.9% in the mBCC and laBCC cohorts, respectively), consistent with the primary analysis.

### Investigator-assessed efficacy

Eight patients in the laBCC cohort were excluded from the efficacy analysis because the independent pathologist did not identify BCC in biopsy specimens taken at baseline or at the post-baseline biopsy. No patients with mBCC were excluded. In the mBCC cohort, the investigator-assessed ORR was 48.5% (95% confidence interval [CI], 30.8–66.2) in this analysis, compared with 45.5% at the primary analysis. All responders in the mBCC cohort achieved a PR per RECIST. Among patients with laBCC, the investigator-assessed ORR was 60.3% (95% CI, 47.2–71.7) in this analysis, comparable with the primary analysis (Table 2). Of the 38 responders in the laBCC cohort, 20 achieved CR and 18 had PR. In general, investigator-assessed ORRs were similar across patient subgroups, although slightly lower response rates were observed in patients with larger tumors (>4 cm), whereas numerically higher response rates were observed in patients aged <65 years and in patients with laBCC from regions outside the United States (Additional file 1: Table S1). Investigator-assessed ORRs were also comparable across histologic subtypes (assessments at baseline by an independent pathologist) (Additional file 1: Table S1). Importantly, clinical effectiveness was demonstrated in aggressive histologic subtypes (eg, ORR of 53.8% and 85.7% in infiltrative laBCC and mBCC, respectively). Investigator-assessed ORR was also evaluated against the number of vismodegib doses missed on study. ORRs observed between patients with no missed doses and patients who missed up to 33% of vismodegib doses were 60.0% (6 out of 10) versus 43.5% (10 out of 23), respectively, in the mBCC cohort and 58.3% (7 out of 12) versus 63.3% (31 out of 49), respectively, in the laBCC cohort (Additional file 1: Table S1). Only 2 efficacy-evaluable patients (both in the laBCC cohort) missed more than 33% of vismodegib doses.

**Table 2:** INV-assessed response, DOR, and PFS

Outcome	Primary analysis (9 months after completion of accrual)		Long-term analysis (39 months after completion of accrual)	
	mBCC	laBCC	mBCC	laBCC
	(*n* = 33)	(*n* = 63)	(*n* = 33)	(*n* = 63)
Objective response, *n* (%) [95% CI]	15 (45.5) [28.1–62.2]	38 (60.3) [47.2–71.7]	16 (48.5) [30.8–66.2]	38 (60.3) [47.2–71.7]
Complete response	0	20	0	20
Partial response	15	18	16	18
Stable disease	15	15	14	15
Progressive disease	2	6	2	6
Median DOR, mo [95% CI] Number of responders	12.9 [5.6–12.9] 15	7.6 [7.4–NE] 38	14.8 [5.6–17.0] 16	26.2 [9.0–37.6] 38
Median PFS, mo [95% CI] Number of events, n	9.2 [7.4–NE] 17	11.3 [9.5–16.8] 26	9.3 [7.4–16.6] 24	12.9 [10.2–28.0] 34
Median OS, mo [95% CI] Number of events, n	NE [13.9–NE] 7	NE [17.6–NE] 6	33.4 [18.1–NE] 17	NE [NE] 13
1-year survival rate, % [95% CI]	75.5 [57.3–93.6]	91.6 [83.5–99.7]	78.7 [64.7–92.7]	93.2 [86.8–99.6]
2-year survival rate, % [95% CI]	NE	NE	62.3% [45.4–79.3]	85.5% [76.1–94.8]

The 95% CI for response rate was calculated using the Blyth-Still-Casella method

*CI* confidence interval, *DOR* duration of response, *INV* investigator, *laBCC* locally advanced basal cell carcinoma, *mBCC* metastatic basal cell carcinoma, *NE* not estimable, *OS* overall survival, *PFS* progression-free survival

**Fig. 1:**
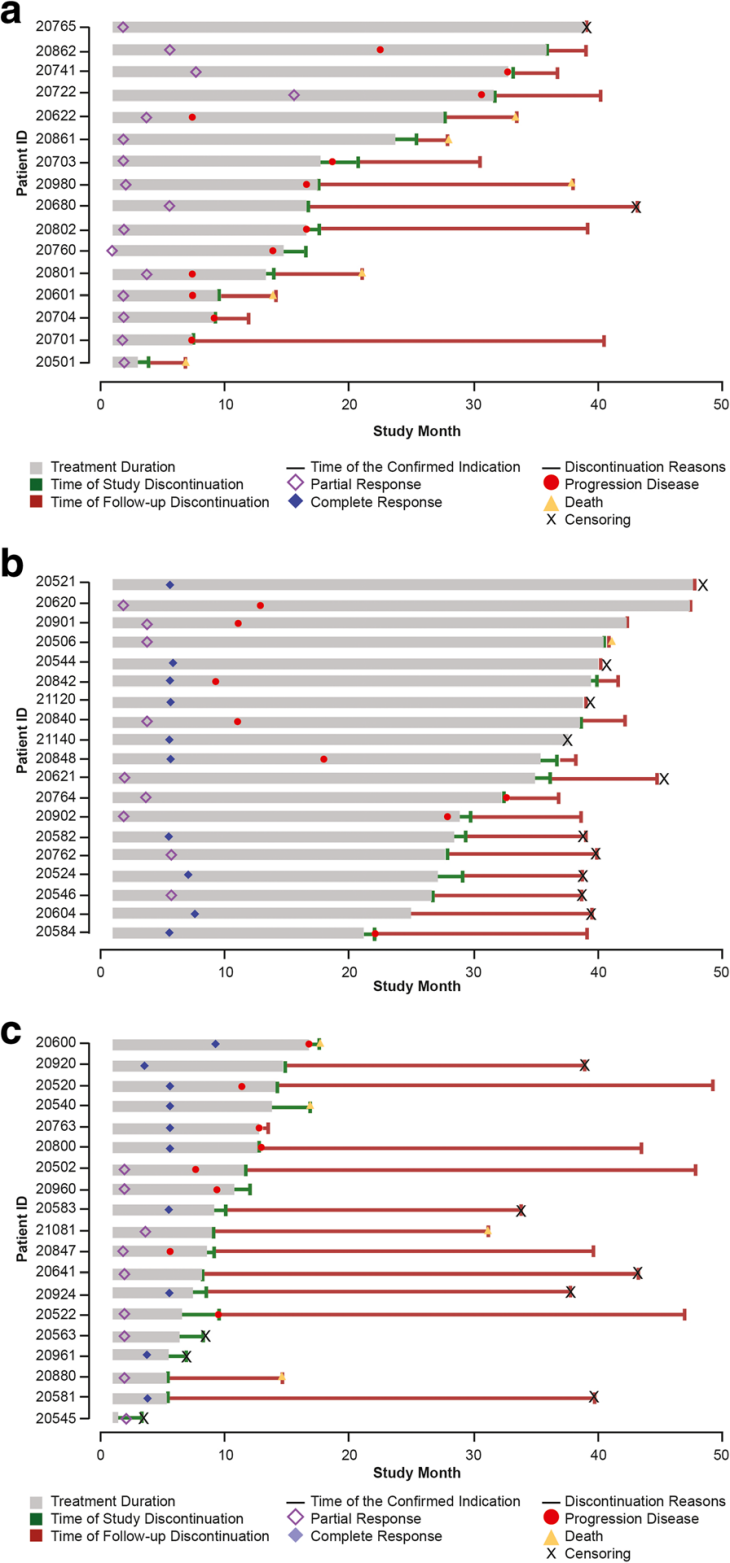
Swimlane plot of time to response, treatment duration, and duration of follow-up for efficacy-evaluable patients who achieved response in the mBCC cohort (**a**) and the laBCC cohort (**b**). laBCC, locally advanced basal cell carcinoma; mBCC, metastatic basal cell carcinoma

**Fig. 2:**
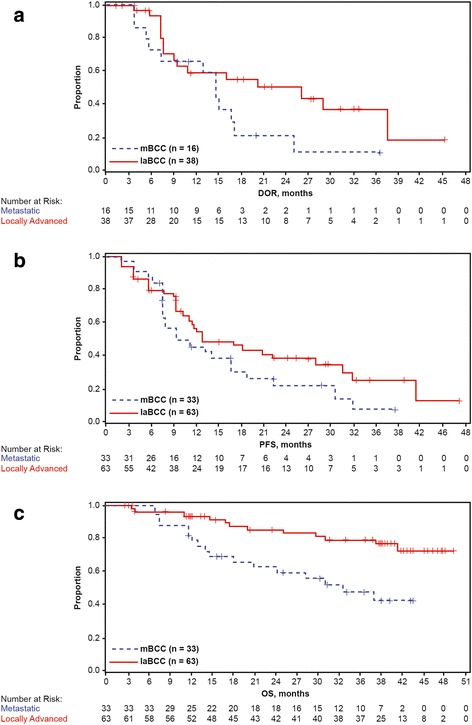
Kaplan–Meier plots of DOR (**a**), PFS (**b**), and OS (**c**) by investigator assessment. DOR, duration of response; laBCC, locally advanced basal cell carcinoma; mBCC, metastatic basal cell carcinoma; OR, overall survival; PFS, progression-free survival

Median time to overall response was 57.0 days (range, 29–473) in the mBCC cohort and 140.0 days (range, 55–281) in the laBCC cohort. Time to response, treatment duration, and duration of follow-up for responders are shown in Fig. 1. Among responders, there was substantial treatment duration (median treatment duration of 17.2 months; range, 1.3–47.8 months), and some responders experienced substantial treatment-free intervals after treatment discontinuation. Estimated median DOR was increased from 12.9 months at the primary analysis (9 months after completion of accrual) to 14.8 months in this final analysis (39 months after completion of accrual) in patients with mBCC. For patients with laBCC, median DOR increased substantially in this period, from 7.6 months to 26.2 months. Kaplan-Meier estimates of DOR by investigator assessment for efficacy-evaluable patients are shown in Fig. 2a. Defining a durable responder (DR) as a patient with DOR greater than the median response duration (ie, >14.8 months for patients with mBCC or >26.2 months for patients with laBCC), a higher proportion of DRs were ECOG PS 0 (80% vs. 55% non-DR patients) and female (52% vs. 38%). No other differences in baseline characteristics between DR and non-DR patients were otherwise apparent.

At the time of data cutoff (39 months after completion of accrual), 24 of 33 efficacy-evaluable patients with mBCC had progressed (by investigator assessment) or died within 30 days of the last treatment. The median investigator-assessed PFS was 9.3 months (95% CI, 7.4–16.6) for those with mBCC and 12.9 months (95% CI, 10.2–28.0) for those with laBCC. Kaplan-Meier estimates of median PFS had increased by 1.7 months from the primary analysis to this final analysis, with increases observed in both cohorts (Table 2 and Fig. 2b).

At this final data cutoff, 30 efficacy evaluable patients had died: 17 of 33 patients (51.5%) with mBCC and 13 of 63 patients (20.6%) with laBCC. Estimated median OS was 33.4 months for the mBCC group but was not estimable for the laBCC group, given the higher survival rate in these patients (Fig. 2c). Median follow-up duration for OS was 39.1 months in both cohorts. Kaplan-Meier estimated survival rates at 1 year were 78.7% (95% CI, 64.7–92.7) and 93.2% (95% CI, 86.8–99.6) in the mBCC and laBCC cohorts, respectively, with both rates showing improvement since the primary analysis (Table 2). The 2-year survival rate was 62.3% (95% CI, 45.4–79.3) in the mBCC cohort and 85.5% (95% CI, 76.1–94.8) in the laBCC cohort.

### Safety

All patients experienced ≥1 TEAE. Although the incidence of TEAEs increased between the time of the primary analysis and this final data cutoff date, most changes in AE incidence were <5% (absolute changes), except for weight decrease and fatigue (increased by 5.9% and 7.3%, respectively). There were no additional occurrences of squamous cell carcinoma (SCC), hypogeusia, ageusia, or amenorrhea (in women of childbearing potential) between the data cutoff dates for the primary analysis and this final update.

The most common TEAEs of any grade (by Medical Dictionary for Regulatory Activities-preferred term) were muscle spasms (71.2%), alopecia (66.3%), dysgeusia (55.8%), weight decreased (51.9%), fatigue (43.3%), and nausea (32.7%) (Table 3). Overall, grade ≥ 3 AEs were reported in 58 patients (55.8%). The most frequent grade ≥ 3 AE was weight decrease (8.7%), followed by muscle spasms (5.8%). Other grade ≥ 3 AEs, including fatigue, decreased appetite, diarrhea, and SCC, occurred in <5% of patients.

**Table 3:** Most common TEAEs by grade

TEAE occurring in >10% of patients, *n* (%)^a^	NCI CTCAE grade (*n* = 104)					
	Total	1	2	3	4	5
Any AE	104 (100.0)	8 (7.7)	37 (35.6)	37 (35.6)	13 (12.5)	8 (7.7)
Muscle spasms	74 (71.2)	45 (43.3)	23 (22.1)	6 (5.8)	0	0
Alopecia	69 (66.3)	49 (47.1)	20 (19.2)	NA	NA	NA
Dysgeusia	58 (55.8)	32 (30.8)	26 (25.0)	NA	NA	NA
Weight decreased	54 (51.9)	29 (27.9)	16 (15.4)	9 (8.7)	NA	NA
Fatigue	45 (43.3)	33 (31.7)	7 (6.7)	4 (3.8)	1 (1.0)	0
Nausea	34 (32.7)	25 (24.0)	9 (8.7)	0	0	0
Decreased appetite	29 (27.9)	19 (18.3)	7 (6.7)	3 (2.9)	0	0
Diarrhea	28 (26.9)	20 (19.2)	5 (4.8)	3 (2.9)	0	0
Constipation	20 (19.2)	14 (13.5)	6 (5.8)	0	0	0
Cough	20 (19.2)	16 (15.4)	4 (3.8)	0	NA	NA
Vomiting	18 (17.3)	15 (14.4)	3 (2.9)	0	0	0
Arthralgia	17 (16.3)	12 (11.5)	4 (3.8)	1 (1.0)	0	0
Headache	15 (14.4)	12 (11.5)	3 (2.9)	0	NA	NA
Nasopharyngitis	13 (12.5)	11 (10.6)	2 (1.9)	0	0	0
SCC	12 (11.5)	3 (2.9)	5 (4.8)	3 (2.9)	0	0
Ageusia	12 (11.5)	8 (7.7)	4 (3.8)	NA	NA	NA
Hypogeusia	11 (10.6)	10 (9.6)	1 (1.0)	NA	NA	NA
Pruritus	11 (10.6)	8 (7.7)	2 (1.9)	1 (1.0)	NA	NA
Dyspepsia	11 (10.6)	8 (7.7)	3 (2.9)	0	NA	NA

*AE* adverse event, *NA* not applicable, *NCI CTCAE* National Cancer Institute Common Terminology Criteria for Adverse Events, version 3.0, *SCC* squamous cell carcinoma, *TEAE* treatment-emergent adverse event

^a^Medical Dictionary for Regulatory Activities–preferred term

SAEs were reported in 36 patients (34.6%) and were considered related to vismodegib in 9 patients (8.7%). SAEs included pneumonia and syncope (4 patients each, 3.8%), hip fracture and death (3 patients each, 2.9%), cardiac failure, cellulitis, gastrointestinal hemorrhage, SCC, pulmonary embolism, and deep vein thrombosis (2 patients each, 1.9%). Importantly, there were contributing factors, including medical history and risk factors, or concurrent AEs that confounded the assessment of the relationship between some SAEs and vismodegib. None of the deaths were considered related to vismodegib treatment by the investigator.

### Incidence of TEAEs according to treatment duration

In general, the incidence of TEAEs was higher in patients with ≥12 months of exposure to vismodegib (*n* = 56) compared to patients with <12 months of treatment exposure (*n* = 48) (Table 4). Patients who received treatment for ≥12 months had higher rates of muscle spasms, alopecia, dysgeusia, weight decreased, fatigue, and nausea than those who received vismodegib for <12 months. Importantly, the overall incidence of grade ≥ 3 TEAEs was similar between patients who received vismodegib for ≥12 months and those who received vismodegib for <12 months (55.4% and 56.3% of patients, respectively). An additional analysis of AEs (all grades and grade ≥ 3) per 100 patient–years of exposure to vismodegib found that the rate of AEs was generally higher during the first year of vismodegib exposure than subsequently (Additional file 2: Table S2). This indicates that, although the incidence of TEAEs was higher in patients who received vismodegib for ≥12 months, the risk of a new AE is reduced after the first year of treatment.

**Table 4:** Most common TEAEs according to duration of exposure to vismodegib

TEAE Occurring in >20% of Patients, *n* (%)^a^	Exposure <12 Months (*n* = 48)		Exposure ≥12 Months (*n* = 56)	
	Any Grade	Grade ≥ 3^b^	Any Grade	Grade ≥ 3^b^
Any AE	48 (100.0)	27 (56.3)	56 (100.0)	31 (55.4)
Muscle spasms	25 (52.1)	2 (4.2)	49 (87.5)	4 (7.1)
Alopecia	24 (50.0)	NA	45 (80.4)	NA
Dysgeusia	20 (41.7)	NA	38 (67.9)	NA
Weight decreased	18 (37.5)	0	36 (64.3)	9 (16.1)
Fatigue	17 (35.4)	4 (8.3)	28 (50.0)	1 (1.8)
Nausea	11 (22.9)	0	23 (41.1)	0
Decreased appetite	15 (31.3)	2 (4.2)	14 (25.0)	1 (1.8)
Diarrhea	10 (20.8)	0	18 (32.1)	3 (5.4)
Constipation	10 (20.8)	0	10 (17.9)	0
Cough	8 (16.7)	0	12 (21.4)	0
Arthralgia	5 (10.4)	0	12 (21.4)	1 (1.8)

*AE* adverse event, *NA* not applicable, *TEAE* treatment-emergent adverse event

^a^Medical Dictionary for Regulatory Activities–preferred term

^b^NCI CTCAE, National Cancer Institute Common Terminology Criteria for Adverse Events, version 3.0

### Deaths

At this data cutoff, 33 deaths (31.7%) due to any cause had been reported (compared with 16 [15.4%] in the primary analysis). The most common causes of death included progressive disease (17 patients, 16.3%) and AEs (8 patients, 7.7%; unrelated to vismodegib based on assessment by the investigator). Of the 17 deaths reported in this update period, only 1 was the result of an AE (general physical health deterioration, considered unrelated to vismodegib). All deaths occurred during survival follow-up (off vismodegib), and none of the additional deaths were considered by the investigator to be related to vismodegib.

## Discussion

Patients with advanced BCC represent a population with significant unmet medical need, particularly those for whom standard treatments (surgery or radiation) are ineffective or would result in unacceptable disfigurement or morbidity from surgery. Vismodegib, an oral, selective HPI, is the first FDA- and EMA-approved HPI and the first drug to be approved for the treatment of advanced BCC. Long-term efficacy and safety of vismodegib are therefore of particular clinical interest.

The results of this study significantly expand upon previous analyses [17, 20] and support the efficacy and durability of response with long-term vismodegib therapy. Clinically relevant data include efficacy demonstrated by investigator-assessed ORR across a range of patient subgroups, not reported previously, including aggressive histologic subtypes (eg, infiltrative BCC). Importantly, efficacy did not appear to be substantially influenced by missed doses of vismodegib. The observation that effectiveness persists in the face of missed doses is important, as some patients do require treatment breaks during long-term treatment to manage AEs and avoid permanent discontinuation. Overall, median duration of treatment on vismodegib was 12.9 months, with some patients continuing on treatment with vismodegib for more than 3 years. Longer treatment duration allows patients to achieve higher total dosing. An exploratory analysis of the STEVIE study also demonstrated that increased treatment interruption was associated with increased median treatment duration and overall response rate [21]. This, along with other factors (such as slight baseline heterogeneity between patients) may, in turn, contribute to the slightly increased response rate observed in laBCC patients who missed up to 33% of doses compared to those who did not miss any doses. Estimated median DOR more than tripled in the laBCC cohort compared with the primary analysis, from 7.6 to 26.2 months, primarily because of prolonged responses over the longer-term follow-up. Interestingly, a number of responders experienced substantial treatment-free intervals after treatment discontinuation. The median OS of 33.4 months in the vismodegib-treated mBCC cohort is particularly significant when compared with the median OS of 24 months in a historical cohort of patients before the availability of vismodegib [22] and a median OS of 8 months in a review of 5 patient cases and 170 published patient cases [23].

The safety profile of vismodegib remained consistent with that reported in the primary analysis. The most common AEs were muscle spasm, dysgeusia, and alopecia, which appear to be class effects associated with on-target inhibition of the Hedgehog signaling pathway [24]. The incidence of these AEs generally increased with longer durations of exposure to vismodegib, with muscle spasms and alopecia occurring in most (>80%) patients who received vismodegib for ≥12 months. There were no additional occurrences of SCC since the primary analysis report, and the incidence of SCC did not differ according to duration of treatment exposure (≥12 months vs <12 months). The percentage of patients who discontinued treatment because of an AE was 21.2% (*n* = 22). Muscle spasms were reported in 5 patients (4.8%) and weight decreased and dysgeusia were each reported in 2 patients (1.9%); all other AEs leading to discontinuation of study drug were reported in 1 patient each. AEs associated with vismodegib treatment are typically grade 1 to 2 in severity; however, the cumulative and chronic nature of these AEs may result in patient discontinuation. A comparable safety profile was observed when vismodegib was assessed in a setting representative of routine clinical practice (STEVIE) where, in an interim analysis, the most common AEs included muscle spasms (64%), alopecia (62%), dysgeusia (54%), and weight loss (33%), with most AEs being grade 1 or 2 in severity [25]. Strategies to manage AEs during long-term vismodegib treatment are essential in order to enable patients to stay on treatment and consequently receive its full benefit. While such management strategies are used in clinical practice (eg, calcium channel blockade or cyclobenzaprine to manage muscle spasms [26]), treatment interruption is a commonly used strategy that allows patients to remain on treatment, without appearing to substantially affect efficacy [21]. Based on the AE analysis per patient–years of vismodegib exposure, which indicates that the risk of a new AE is higher during the first year of treatment, such management strategies are particularly important during this period.

## Conclusions

In conclusion, this long-term study of vismodegib involving 39 months of observation after the completion of accrual in the ERIVANCE BCC trial reinforces the clinical usefulness of vismodegib in patients with advanced BCC for whom treatment options are limited, and demonstrates the durability of response and long-term safety of vismodegib.

## Additional files

Additional file 1: Table S1. Investigator-assessed response across patient subgroups (efficacy-evaluable patients). Assessments and demographics for patients with metastatic BCC and locally advanced BCC. (DOCX 16 kb)

Additional file 2: Table S2. Most common TEAEs per 100 patient-years of exposure to vismodegib. TEAEs by type during vismodegib treatment for 1 year and after 12 months’ treatment. (DOCX 14 kb)

## Abbreviations

AE: Adverse event
BCC: Basal cell carcinoma
BCCNS: Basal cell carcinoma nevus syndrome
CI: Confidence interval
CR: Complete response
DOR: Duration of response
ECOG: Eastern Cooperative Oncology Group
EMA: European Medicines Agency
FDA: US Food and Drug Administration
HPI: Hedgehog pathway inhibitor
IRF: Independent review facility
laBCC: Locally advanced basal cell carcinoma
mBCC: Metastatic basal cell carcinoma
ORR: Objective response rate
OS: Overall survival
PFS: Progression-free survival
PR: Partial response
RECIST: Response evaluation criteria in solid tumors
SAE: Serious adverse event
SCC: Squamous cell carcinoma
TEAE: Treatment-emergent adverse event
