# Correction to: Long-term safety and efficacy of vismodegib in patients with advanced basal cell carcinoma: final update of the pivotal ERIVANCE BCC study

**DOI:** 10.1186/s12885-019-5568-6

**Published:** 2019-04-18

**Authors:** Aleksandar Sekulic, Michael R. Migden, Nicole Basset-Seguin, Claus Garbe, Anja Gesierich, Christopher D. Lao, Chris Miller, Laurent Mortier, Dedee F. Murrell, Omid Hamid, Jorge F. Quevedo, Jeannie Hou, Edward McKenna, Natalie Dimier, Sarah Williams, Dirk Schadendorf, Axel Hauschild, for the ERIVANCE BCC Investigators

**Affiliations:** 1 Mayo Clinic Scottsdale, 13400 East Shea Boulevard, Scottsdale, AZ 85259 USA; 2 Departments of Dermatology and Head and Neck Surgery, The University of Texas MD Anderson Cancer Center, 1400 Pressler Street, Houston, TX 77030 USA; 3 Service de Dermatologie, Hôpital Saint-Louis, 1 av claude Vellefaux, 75010 Paris, France; 4 Studienzentrum Dermatologische Onkologie, Universitätsklinikum Tübingen, Liebermeisterstr. 25, 72074 Tübingen, Germany; 5 Klinik für Dermatologie, Venerologie und Allergologie, Universitätsklinikum Würzburg, Josef-Schneider-Str. 2, 97080 Würzburg, Germany; 6 University of Michigan, 1500 East Medical Center Drive, Ann Arbor, MI 48109 USA; 7 Department of Dermatology, University of Pennsylvania Medical Center, 3400 Civic Center Boulevard, Philadelphia, PA 19104 USA; 8 Clinique de Dermatologie, Hôpital Claude Huriez, Inserm U1189, Lille, France; 9 Dermatology Department, St George Clinical School, University of New South Wales, Grey Street, Sydney, 2217 Australia; 10 The Angeles Clinic and Research Institute, 1818 Wilshire Boulevard, California, Los Angeles USA; 11 Mayo Clinic, 200 First Street SW, Rochester, MN 55905 USA; 12 Genentech, Inc., 1 DNA Way, South San Francisco, California 94080 USA; 13 Roche Products Limited, Hexagon Place, 6 Falcon Way, Shire Park, Welwyn Garden City, Hertfordshire Al7 1TW UK; 14 Klinikum für Dermatologie, Venerologie und Allergologie, Universitätsklinikum Essen, Hufelandstrabe 55, 45147 Essen, Germany; 15 Universitätsklinikum Schleswig-Holstein, Schittenhelmstr, 7, D-24 105 Kiel, Germany

**Correction to: BMC Cancer**

**DOI 10.1186/s12885-017-3286-5**

Following publication of the original article [1], it was reported that the legend for Fig. 1 was incomplete. The complete figure legend is:

**Fig. 1** Swimlane plot of time to response, treatment duration, and duration of follow-up for efficacy-evaluable patients who achieved response in the mBCC cohort (**a**), those in the laBCC cohort with treatment duration > 20 months (**b**), and those in the laBCC cohort who were treated for < 20 months (**c**). laBCC, locally advanced basal cell carcinoma; mBCC, metastatic basal cell carcinoma.
