# Supplementary material for: Long-term safety and efficacy of vismodegib in patients with advanced basal cell carcinoma: final update of the pivotal ERIVANCE BCC study
Source: BMC Cancer. 2017 May 16;17:332. doi: 10.1186/s12885-017-3286-5 (PMC5433030; doi:10.1186/s12885-017-3286-5)
Supplement: Supplementary file 1 — Investigator-assessed response across patient subgroups (efficacy-evaluable patients). Assessments and demographics for patients with metastatic BCC and locally advanced BCC. (DOCX 16 kb) [file 12885_2017_3286_MOESM1_ESM.docx]

**Supplementary Table S1.** Investigator-assessed response across patient subgroups (efficacy-evaluable patients)

|  | | **mBCC** | | **laBCC** | | **All patients** | |
| --- | --- | --- | --- | --- | --- | --- | --- |
|  | ***N*** | | **Response, *n* (%)** | ***N*** | **Response, *n* (%)** | ***N*** | **Response, *n* (%)** |
| ECOG PS |  | |  |  |  |  |  |
| 0 | 13 | | 6 (46.2) | 48 | 30 (62.5) | 61 | 36 (59.0) |
| 1 | 19 | | 10 (52.6) | 13 | 8 (61.5) | 32 | 18 (56.3) |
| 2 | 1 | | 0 | 2 | 0 | 3 | 0 |
| Age |  | |  |  |  |  |  |
| <65 years | 19 | | 11 (57.9) | 33 | 24 (72.4) | 52 | 35 (67.3) |
| ≥65 years | 14 | | 5 (35.7) | 30 | 14 (46.7) | 44 | 19 (43.2) |
| Region |  | |  |  |  |  |  |
| US | 26 | | 13 (50.0) | 42 | 23 (54.8) | 68 | 36 (52.9) |
| Non-US | 7 | | 3 (42.9) | 21 | 15 (71.4) | 28 | 18 (64.3) |
| Sex |  | |  |  |  |  |  |
| Male | 24 | | 11 (45.8) | 35 | 19 (54.3) | 59 | 30 (50.8) |
| Female | 9 | | 5 (55.6) | 28 | 19 (67.9) | 37 | 24 (64.9) |
| Number of target lesions at baseline |  | |  |  |  |  |  |
| 1 | 9 | | 2 (22.2) | 40 | 24 (60.0) | 49 | 26 (53.1) |
| 2 | 4 | | 1 (25.0) | 12 | 6 (50.0) | 16 | 7 (43.8) |
| 3 | 9 | | 5 (55.6) | 6 | 5 (83.3) | 15 | 10 (66.7) |
| > 3 | 11 | | 8 (72.7) | 5 | 3 (60.0) | 16 | 11 (68.8) |
| Mean tumor size at baseline^a^ |  | |  |  |  |  |  |
| <2 cm | 7 | | 4 (57.1) | 6 | 5 (83.3) | 13 | 9 (69.2) |
| 2–3 cm | 17 | | 8 (47.1) | 15 | 11 (73.3) | 32 | 19 (59.4) |
| 3–4 cm | 4 | | 2 (50.0) | 9 | 6 (66.7) | 13 | 8 (61.5) |
| >4 cm | 5 | | 2 (40.0) | 32 | 16 (50.0) | 37 | 18 (48.6) |
| Histologic subtype |  | |  |  |  |  |  |
| Infiltrative | 7 | | 6 (85.7) | 39 | 21 (53.8) | 46 | 27 (58.7) |
| Micronodular | 1 | | 0 | 8 | 4 (50.0) | 9 | 4 (44.4) |
| Nodular | 0 | | 0 | 12 | 10 (83.3) | 12 | 10 (83.3) |
| Superficial | 0 | | 0 | 1 | 0 | 1 | 0 |
| BCC-NOS | 25 | | 10 (40.0) | 3 | 3 (100.0) | 28 | 13 (46.4) |
| Number of missing doses during study |  | |  |  |  |  |  |
| 0 | 10 | | 6 (60.0) | 12 | 7 (58.3) | 22 | 13 (59.1) |
| <33% | 23 | | 10 (43.5) | 49 | 31 (63.3) | 72 | 41 (56.9) |
| 33–67% | 0 | | - | 2 | 0 | 2 | 0 |

*BCC-NOS* basal cell carcinoma not otherwise specified, *ECOG PS* Eastern Cooperative Oncology Group performance status, *laBCC* locally advanced basal cell carcinoma, *mBCC* metastatic basal cell carcinoma

^a^One patient in the laBCC cohort had no tumor size measurements at baseline
