# Supplementary material for: Long-term safety and efficacy of vismodegib in patients with advanced basal cell carcinoma: final update of the pivotal ERIVANCE BCC study
Source: BMC Cancer. 2017 May 16;17:332. doi: 10.1186/s12885-017-3286-5 (PMC5433030; doi:10.1186/s12885-017-3286-5)
Supplement: Supplementary file 2 — Most common TEAEs per 100 patient-years of exposure to vismodegib. TEAEs by type during vismodegib treatment for 1 year and after 12 months’ treatment. (DOCX 14 kb) [file 12885_2017_3286_MOESM2_ESM.docx]

**Supplementary Table S2.** Most common^a^ TEAEs per 100 patient-years of exposure to vismodegib

| **Number of TEAEs^b^ (rate per 100 patient-years)** | **During vismodegib exposure up to 12 months**  **(81.94 patient-years)** | | **During vismodegib exposure beyond 12 months (64.45 patient-years)** | |
| --- | --- | --- | --- | --- |
|  | **Any grade** | **Grade**  **≥ 3^c^** | **Any grade** | **Grade**  **≥ 3^c^** |
| Total number of patients with at least 1 AE | 104 | 47 | 44 | 24 |
| Overall number of events | 1017 (1241.1) | 87  (106.2) | 243  (377.0) | 48 (74.5) |
| Muscle spasms | 73 (89.1) | 5 (6.1) | 1 (1.6) | 1 (1.6) |
| Alopecia | 67 (81.8) | 0 | 2 (3.1) | 0 |
| Dysgeusia | 56 (68.3) | 0 | 2 (3.1) | 0 |
| Weight decreased | 48 (58.6) | 3 (3.7) | 6 (9.3) | 6 (9.3) |
| Fatigue | 38 (46.4) | 4 (4.9) | 7 (10.9) | 1 (1.6) |
| Nausea | 29 (35.7) | 0 | 5 (7.8) | 0 |
| Decreased appetite | 25 (30.5) | 3 (3.7) | 4 (6.2) | 0 |
| Diarrhea | 23 (28.1) | 1 (1.2) | 5 (7.8) | 2 (3.1) |
| Constipation | 17 (20.1) | 0 | 3 (4.7) | 0 |

Multiple occurrences of a specific TEAE for a patient were counted once at the first occurrence

Numbers in brackets correspond to rates of AEs occurring during each time interval; lower numbers indicate a lower risk of a new AE

*AE* adverse event, *TEAE* treatment-emergent adverse event

^a^≥20 TEAEs in the pre–12 months’ exposure group

^b^Medical Dictionary for Regulatory Activities–preferred term

^c^NCI CTCAE, National Cancer Institute Common Terminology Criteria for Adverse Events, version 3.0
